# Supplementary material for: β-catenin-driven endomesoderm specification is a Bilateria-specific novelty
Source: Nat Commun. 2025 Mar 12;16:2476. doi: 10.1038/s41467-025-57109-w (PMC11903683; doi:10.1038/s41467-025-57109-w)
Supplement: Supplementary file 1 — Supplementary Information [file 41467_2025_57109_MOESM1_ESM.pdf]

SUPPLEMENTARY INFORMATION

β-catenin-driven endomesoderm specification is a Bilateria-specific novelty

Tatiana Lebedeva, Johan Boström, Stanislav Kremnyov, David Mörsdorf, Isabell Niedermoser, Evgeny Genikhovich, Andreas Hejnol, Igor Adameyko, Grigory Genikhovich

Contents

Supplementary Figures 1-8
Supplementary Note 1
Supplementary references
Supplementary Tables 1-2

Supplementary Figures

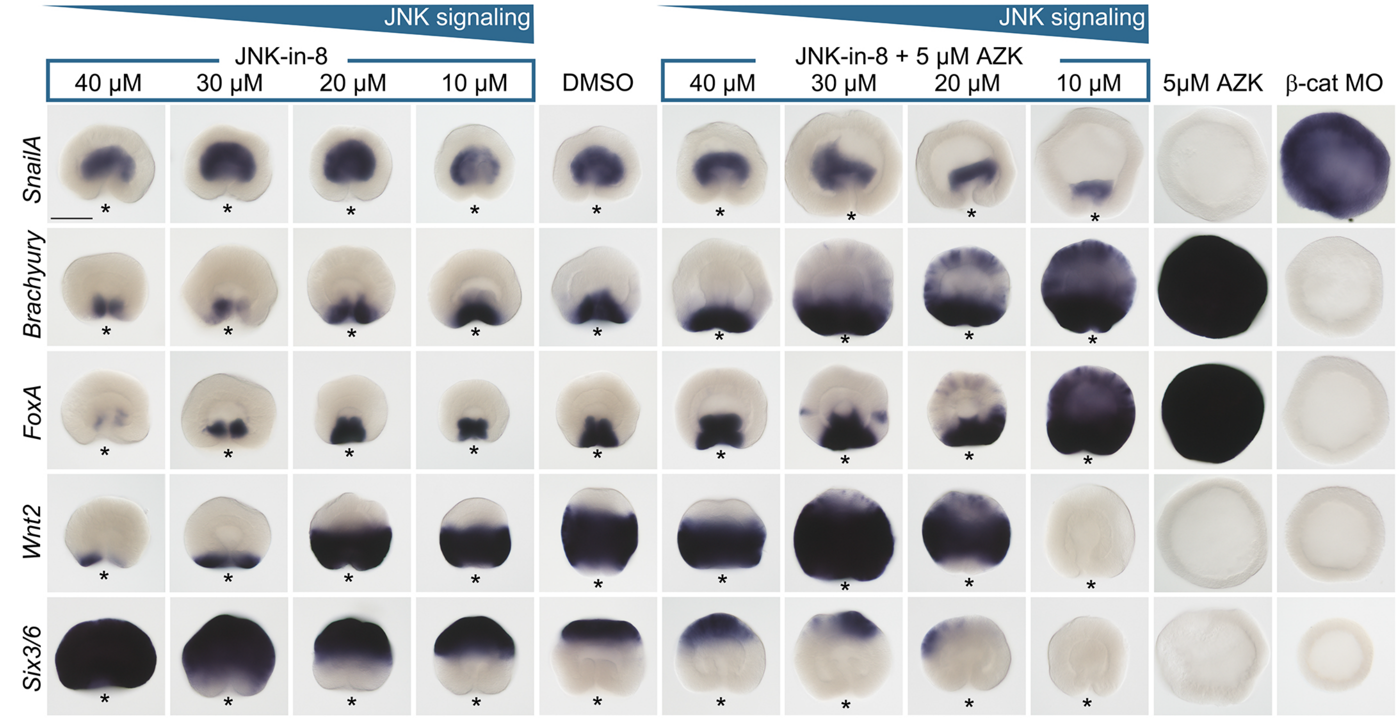

Supplementary Fig. 1. Similar to the situation in sea urchin[26], treatment with the JNK signaling inhibitor JNK-in-8 dose-dependently aboralizes *Nematostella* embryos and rescues oralization caused by β-catenin stabilization with azakenpaullone (AZK). *SnailA* is a mesodermal marker, *Brachyury* and *FoxA* are oral/posterior endodermal markers, *Wnt2* is a midbody ectodermal marker, *Six3/6* is an aboral/anterior ectodermal marker. The effect of the β-catenin morpholino injection is shown for comparison and confirms the previously published data[19,23]. All the embryos are 30 hpf (late gastrula stage in controls). Asterisks mark the position of the blastopore. Scale bar 100 µm. All treatments were replicated three times with similar results.

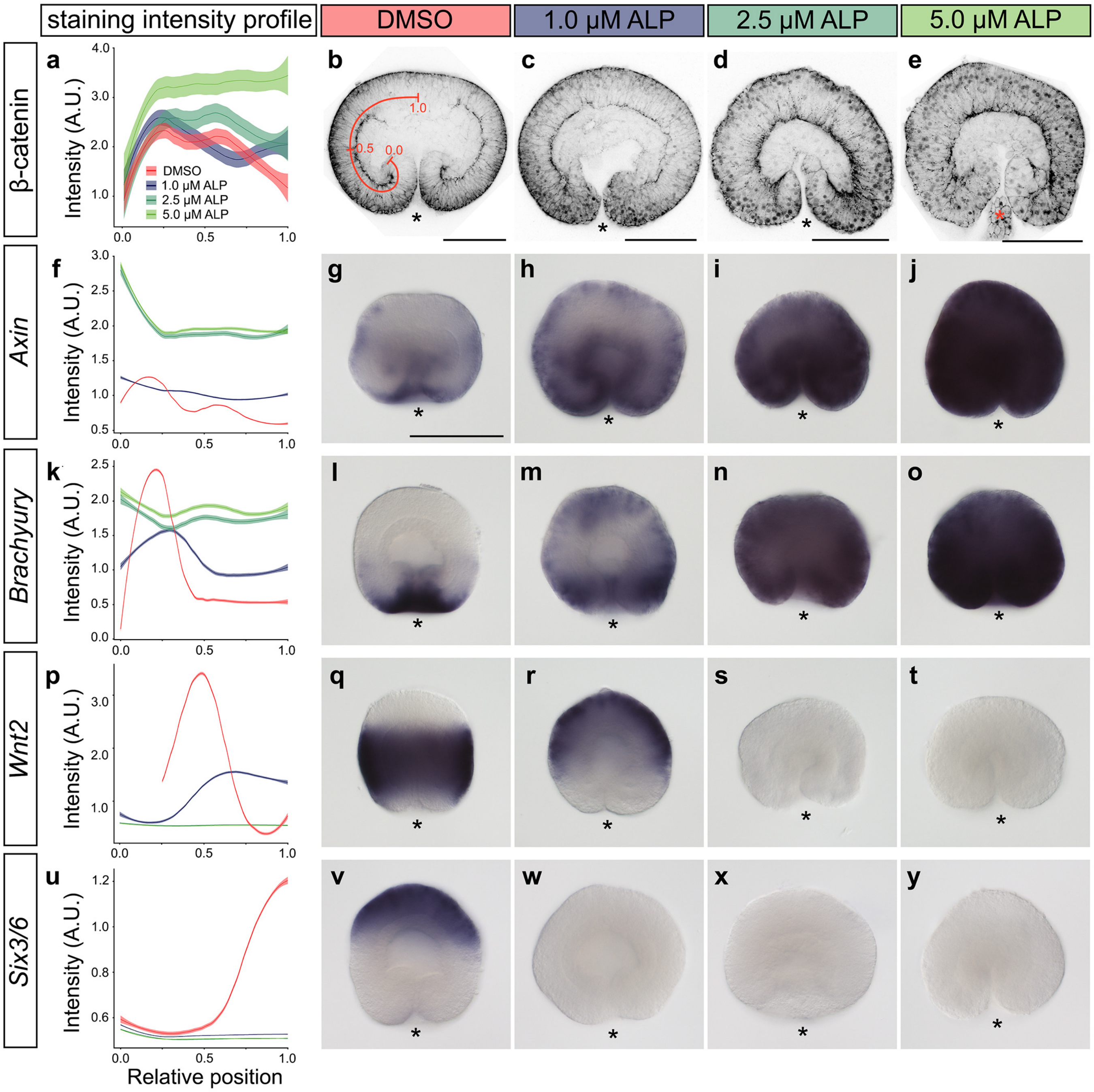

Supplementary Fig. 2. Changes in the expression of the β-catenin-dependent genes upon alsterpaullone (ALP) treatment are in line with the changes to the sfGFP-β-catenin gradient. (a-d) Changes in the amount of nuclear sfGFP-β-catenin. **(f-y)** Changes in the marker gene expression. The measurements of the signal were performed in the endo- and ectoderm from relative position 0 to relative position 1 (see orange line on B). In case of *Wnt2*, staining intensities were not plotted for the DMSO-treated embryos between relative positions 0 and 0.25 because correct measurements are impossible due to strong staining in the overlying ectoderm between relative positions 0.25 and 0.75. First column shows LOESS smoothed curves and 99% confidence intervals for the mean staining intensity. See also Source Data. Scale bar 100 µm. All embryos are 30 hpf late gastrulae. Asterisks mark the blastopore. *Axin* is a sensitive target of β-catenin signaling. *Brachyury* and *FoxA* are oral/posterior endodermal markers, *Wnt2* is a midbody ectodermal marker, *Six3/6* is an aboral/anterior marker. All treatments were replicated three times with similar results.

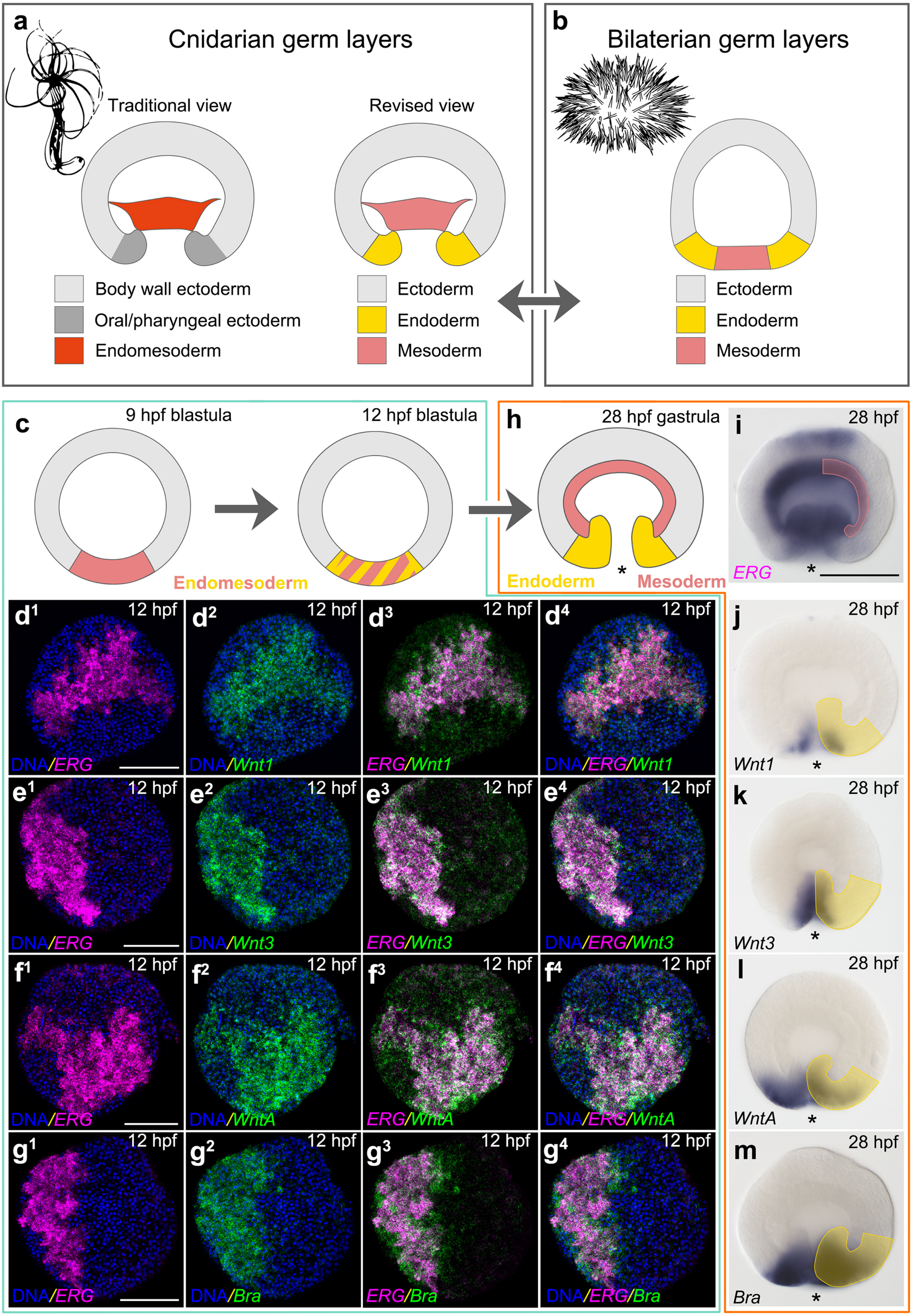

Supplementary Fig. 3 Legend on the next page >>

Supplementary Fig. 3. Germ layer homology between Cnidaria and Bilateria and evidence for cnidarian endomesoderm. (a-b) Views on cnidarian and bilaterian germ layer homology. **(c-g⁴)** Mesodermal marker *ERG* is co-expressed with the endodermal markers *Wnt1*, *Wnt3*, *WntA* and *Bra* in the endomesodermal domain of the *Nematostella* 12 hpf (hours post fertilization) blastula. Note that *Wnt1*, *WntA* and, especially, *Bra* expression expands out of the *ERG* domain into the forming definitive endoderm. Maximum intensity projections are shown; the irregular shape of the endomesodermal domain is typical for normal *Nematostella* development. A sketch showing a 9 hpf blastula on **(c)** indicates that mesodermal gene expression predates the onset of the endodermal genes in the endomesodermal domain. See also [31]. **(h-m)** In late gastrula, *ERG* is strongly expressed in the mesoderm (with additional weaker expression in the endoderm and aboral ectoderm appearing later in development), while *Wnt1*, *Wnt3*, *WntA* and *Bra* are exclusively expressed in the endoderm. Scale bars 100 µm. All stainings were replicated three times with similar results.

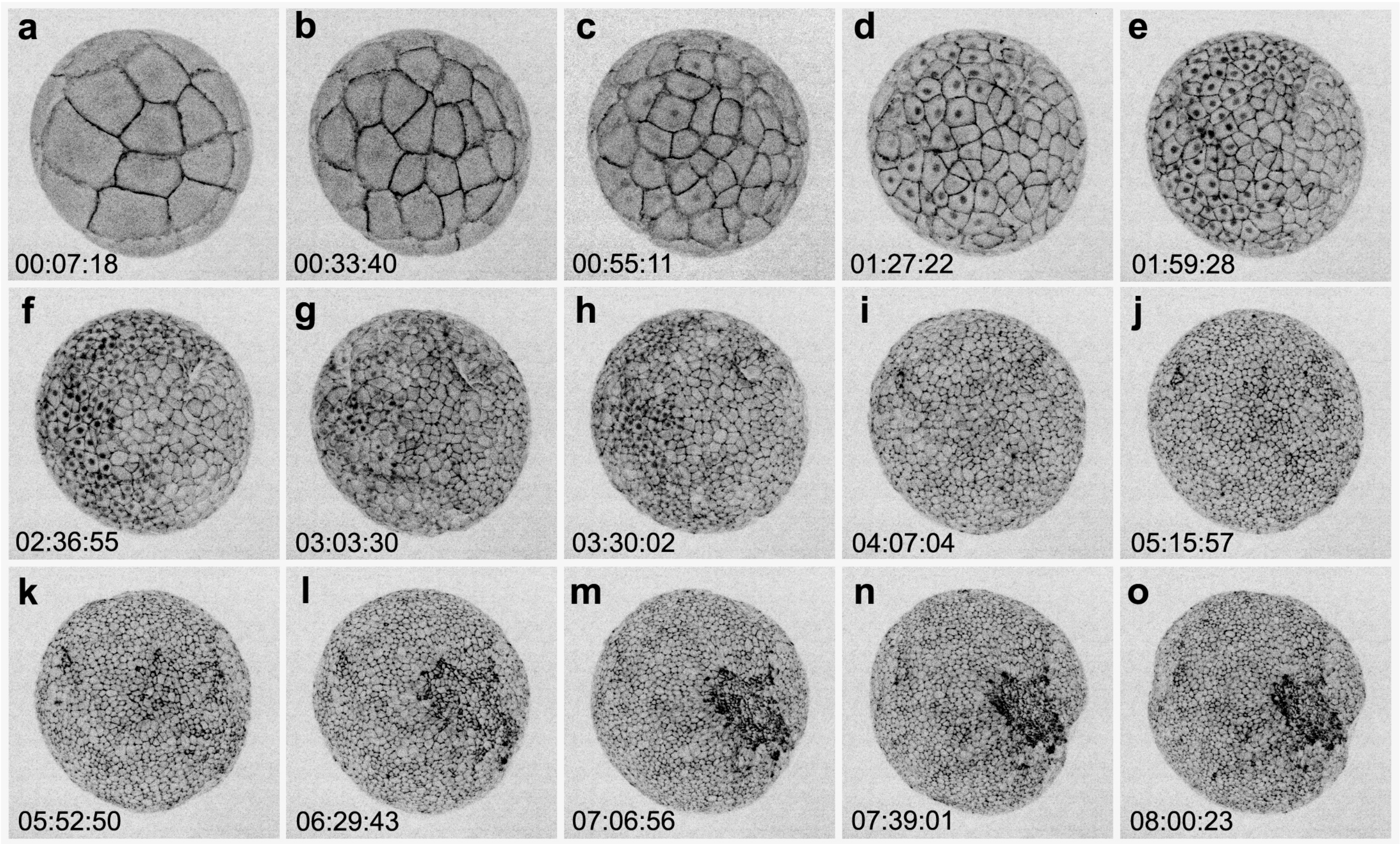

Supplementary Fig. 4. Frames from the Supplementary Movie 1 shown on Fig. 2a-o with actual time stamps. Time format is hh:mm:ss. Time 00:00:00 corresponds to the first frame of the movie, which started when the embryo was in the 32-cell stage.

Frame	Timestamp #	Timestamp in seconds	Time stamp in hh:mm:ss
a	timestamp #02	438.26741134000105	00:07:18
b	timestamp #07	2020.307312140001	00:33:40
c	timestamp #11	3311.2645746999997	00:55:11
d	timestamp #17	5241.94769806	01:27:22
e	timestamp #23	7168.488249580001	01:59:28
f	timestamp #30	9414.748711780001	02:36:55
g	timestamp #35	11010.3850681	03:03:30
h	timestamp #40	12602.30939782	03:30:02
i	timestamp #47	14824.65011758	04:07:04
j	timestamp #60	18957.1284403	05:15:57
k	timestamp #67	21170.40614638	05:52:50
l	timestamp #74	23383.41886582	06:29:43
m	timestamp #81	25616.39117218	07:06:56
n	timestamp #87	27541.028658940002	07:39:01
o	timestamp #91	28823.036654859996	08:00:23

A faster development is due to the temperature difference between the conditions at which average developmental times were determined [32] (18°C) and ~23°C at which the time-lapse was made (see Materials and Methods). Importantly, *Nematostella* development is completely normal at these higher temperatures. We routinely raise embryos at 26°C if we need to reach primary polyp stage quicker.

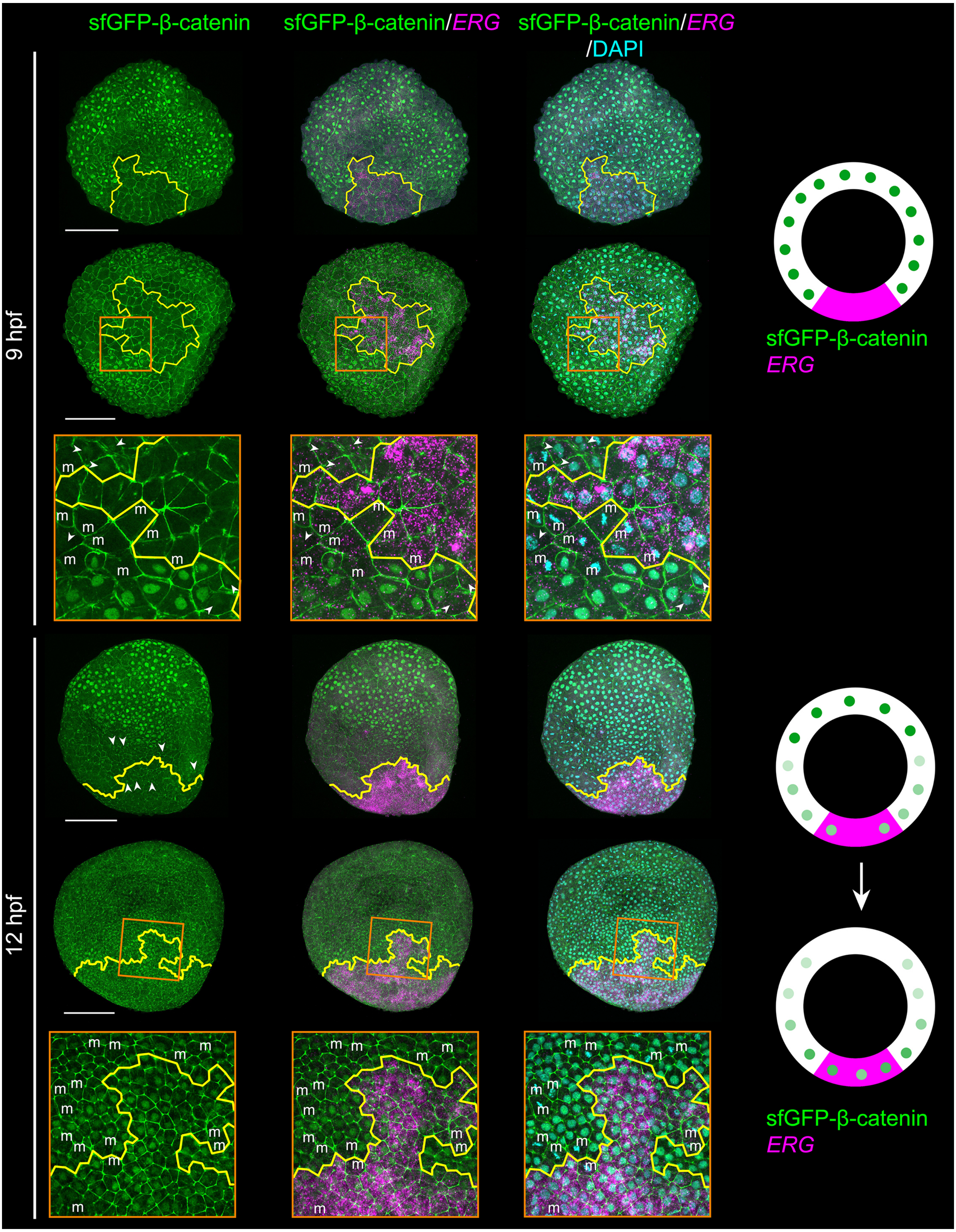

Supplementary Fig. 5 Relative position of the nuclear sfGFP-β-catenin signal and the endomesodermal domain visualized by expression of the early mesodermal marker ERG. HCR with probes against the mesodermal marker *ERG* (magenta) combined with anti-GFP antibody staining. Strong maternal sfGFP-β-catenin signal is observed in

Legend continues on the next page >>

Supplementary Fig. 5 (Continued)

the interphase nuclei of the ERG-negative, ectodermal cells of the 9 hpf embryo. At 12 hpf, strong vegetal sfGFP-β-catenin signal progressively disappears and is gradually replaced by weaker zygotic nuclear sfGFP-β-catenin signal with a maximum in a ring spanning the *ERG* expression boundary. Maximum projections of the z-stacks. Yellow line demarcates the boundary of the endomesoderm (=*ERG*-expressing cells). Orange boxes on the overview images show the areas shown below at higher magnification. Mitotic cells (based on DAPI signal) are indicated on the high magnification images with "m". White arrowheads point at nuclei with weak sfGFP-β-catenin signal. Scale bars 100 µm. Stanings were replicated three times with similar results.

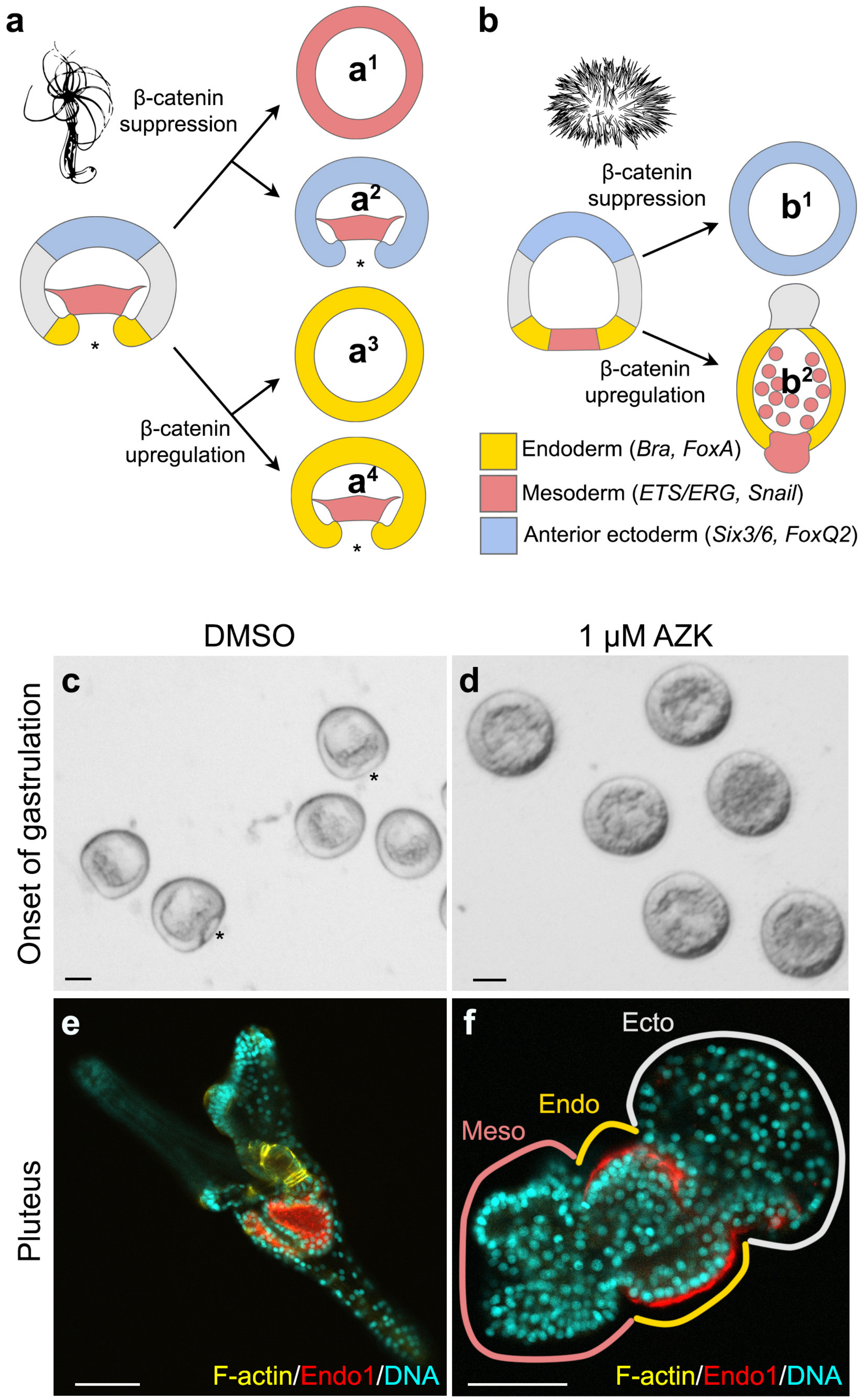

Supplementary Fig. 6. Comparison of the effects of the up- and downregulation of β-catenin signalling in the embryos of the sea anemone and sea urchin. (a-b) Comparison of the effect of the downregulation of β-catenin signalling in *Nematostella* and sea urchin. **(a1)** Effect of the β-catenin morpholino injection. **(a2)** Effect of the knockdown of LRP5/6 or of all four Frizzled receptors. **(a^3)** Effect of early (<6 hpf) β-catenin signalling activation. **(a4)** Effect of late (>6 hpf) β-catenin signalling activation. **(b1)** Effect of *Axin* mRNA injection. **(b2)** Effect of constitutively active β-catenin mRNA injection. The cartoons showing the effects of the up- and downregulation of the β-catenin signalling are based on data from [19,23,26,33]. **(c-d)** Live observation shows that by the time DMSO-treated embryos of the

Legend continues on the next page >>

Supplementary Fig. 6 (Continued)

sea urchin *Paracentrotus lividus* start invaginating (see asterisks on **c**), the blastocoel of the AZK-treated embryos becomes filled with mesenchymal cells, while the embryo stays spherical. **(e-f)** By the time DMSO-treated *Paracentrotus* embryos reach pluteus stage **(e)**, AZK-treated embryos acquire the "snowman" phenotype **(f)** previously described for LiCl-treated embryos[66]. This phenotype is a result of exogastrulation caused by a severe expansion of the endomesodermal domain. Endodermal cells are visualized with the Endo1 antibody (red), mesodermal cells on **(d)** form a folded structure on the bottom left side. Ectodermal cells form a smooth bubble-like structure on the top right. Yellow staining on **(c)** shows muscles surrounding the oesophagus of the pluteus. Scale bars 50 µm. Experiments were replicated five times with similar results.

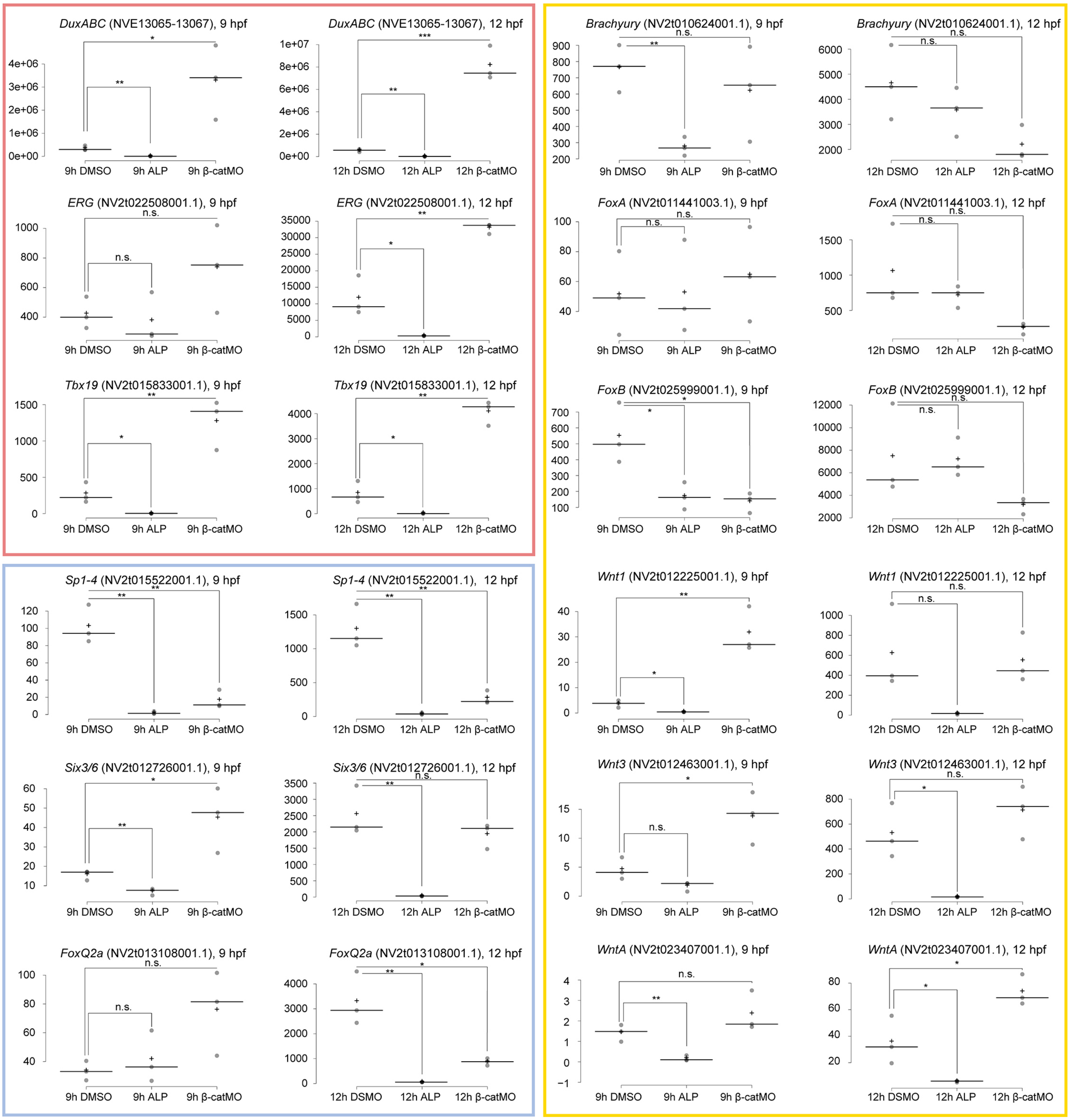

Supplementary Fig. 7. qPCR analysis of the germ layer markers in 9 hpf and 12 hpf embryos upon treatment with GSK3 inhibitor alsterpaullone (ALP) and in β-catenin morphants (β-catMO). Expression intensity values on Y-axis are in arbitrary units, but same Y values reflect same relative gene expression levels across all genes and all experimental conditions (i.e. Y=100 will correspond to the same expression level in all graphs). All tested early mesodermal markers (pink box) are suppressed by ALP treatment and activated in β-catenin morphants suggesting. All tested endodermal markers (yellow box) are known to be positively regulated by Wnt/β-catenin signaling at later developmental stages (e.g. gastrula and planula[15,17–19], and their expression (except for *Brachyury*, which starts at 8 hpf) is detectable by in situ hybridization from 10-12 hpf on[15,31]. Strikingly, none of them is upregulated by ALP or suppressed in β-catenin morphants at the 9 or 12 hpf blastula stage. Moreover, endodermal genes, whose expression is observed in the endomesodermal domain in the blastula (*Bra*, *Wnt1*, *Wnt3*, *WntA* - see Supplementary Fig. 3) appear to be suppressed by ALP treatement at this stage. This suggests that expression of all these markers of the definitive endoderm is initiated by signals other than β-catenin and only subsequently becomes β-catenin-dependent. Three zygotic markers of the aboral/anterior ectoderm (blue box), which normally start to be expressed around 12 hpf, are

Legend continues on the next page >>

Supplementary Fig. 7 (Continued)

all suppressed by ALP treatment at 12 hpf, as is also the case in older embryos. *Sp1-4* and *FoxQ2a* are also downregulated in β-catenin morphants in 12 hpf blastulae, while *Six3/6* is not affected by β-catenin knockdown at this stage. This is in contrast to its suppression in slightly older β-catenin knockdown embryos[23]. Individual datum points are depicted as grey circles (n=3 biological replicates in all experiments), bars show the medians and pluses indicate the means for each condition. Significance was estimated using a two-tailed t-test, however, due to the low sample size the normality of the distribution had to be assumed. * - $p<0.05$, ** - $p<0.01$, *** - $p<0.001$, n.s. - not significant. See Source Data for the plotted data and exact p-values for each condition and Supplementary Note 1 for the discussion.

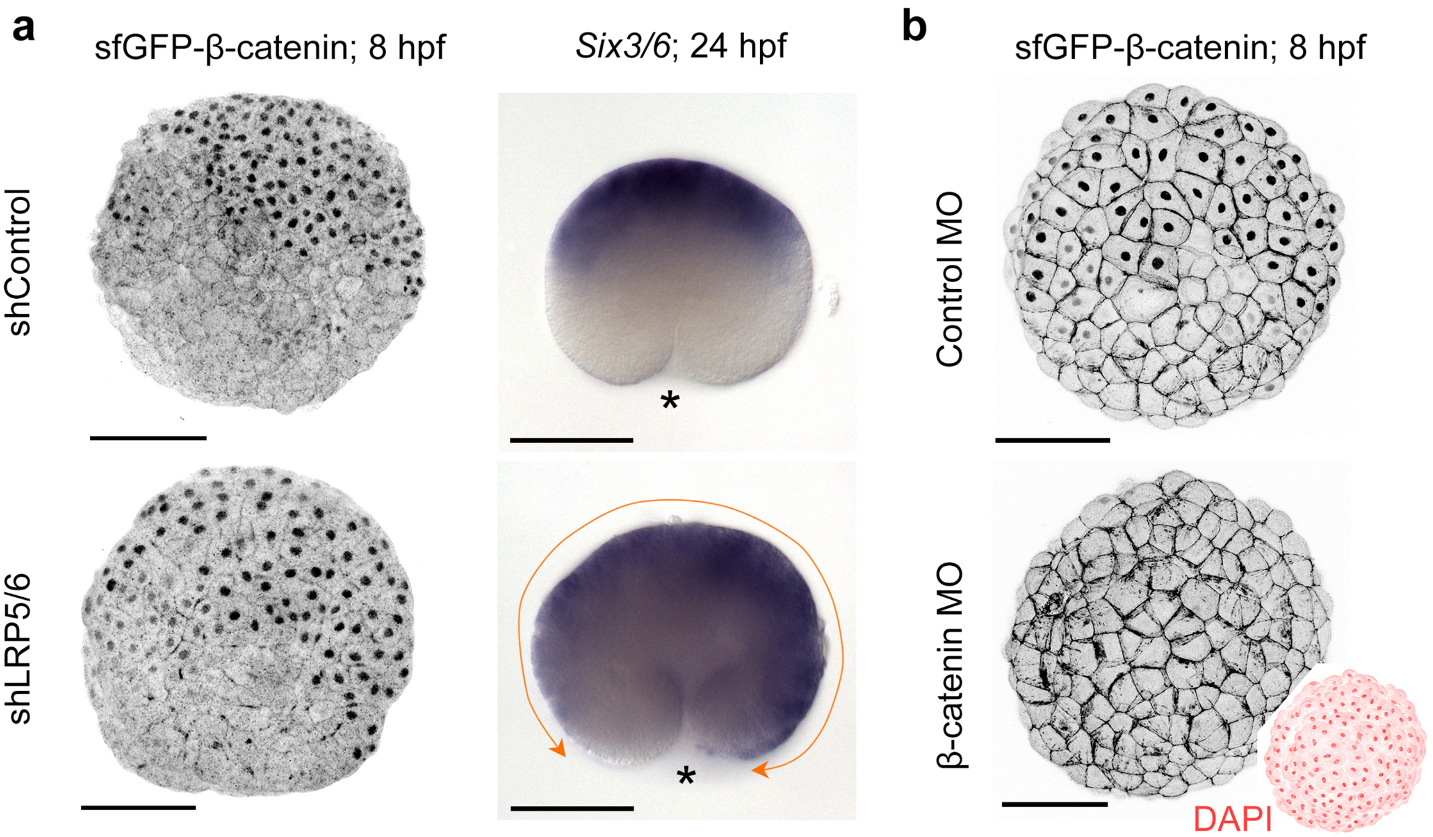

Supplementary Fig. 8. The difference in the knockdown phenotypes caused by RNAi against LRP5/6 and by inhibition of β-catenin translation. (a) RNAi against *Nematostella LRP5/6* does not prevent accumulation of the maternal nuclear sfGFP-β-catenin in the 8 hpf embryo and subsequent gastrulation but causes a previously described axial patterning defect of severe aboralization manifested by expansion of the aboral/anterior ectoderm marker *Six3/6* expression towards the oral end of the gastrula (see [19]). **(b)** In contrast, injection of the translation-blocking morpholino against β-catenin abolishes maternal nuclear sfGFP-β-catenin in the 8 hpf embryo. DAPI staining (inset) shows that although no nuclear sfGFP-β-catenin is observed, all the cells are in the interphase. sfGFP-β-catenin is detected by anti-GFP antibody staining of the embryos developing from eggs laid by the knock-in females homozygous for sfGFP-β-catenin. Images show maximum intensity projections of the confocal z-stacks. Asterisks denote the blastopore. Scale bars 100 µm. The experiments were replicated three times with similar results.

Supplementary Note 1

Results of the down- and upregulation of β-catenin signalling in *Nematostella* and sea urchin

Previous analyses of the *β-catenin-GFP* mRNA injected *Nematostella* embryos showed nuclear β-catenin-GFP localization on one side of the early blastula in the untreated embryos and in all blastoderm cells of the embryos upon GSK3β inhibition [11,15]. Moreover, *Nematostella* embryos with the nuclear localization of β-catenin suppressed either by truncated sea urchin cadherin mRNA overexpression, injection of the β-catenin translation blocking morpholino or β-catenin RNAi failed to gastrulate, and did not express endodermal markers such as *Brachyury*, *FoxA* and *FoxB* remaining blastula-like spheres[11,23,37]. Morphologically, this effect closely resembled the gastrulation block caused by injection of the mRNA encoding truncated cadherin or the DIX domain of Dishevelled in sea urchin[5] and led to the conclusion that the endomesoderm in *Nematostella*, just like the endomesoderm in a number of bilaterians, is specified by an early β-catenin signal at the future gastrulation pole of the embryo[11]. Although universally accepted in the field (also by us – see for example [15,38]), this hypothesis was contradicted by several important observations (Fig. 2l-m). First, in spite of remaining completely spherical and failing to gastrulate, *Nematostella* β-catenin morphants ubiquitously expressed mesodermal marker *SnailA* and other mesodermal markers, rather than the zygotic ectodermal markers of the aboral/anterior/"low β-catenin signalling" end of the OA axis such as *Six3/6* or *FoxQ2a* (Supplementary Fig. 1, Supplementary Fig. 6a[1]; [23,31]). Second, knockdown of the crucial cWnt signalling co-receptor LRP5/6 as well as combined knockdown of all four *Nematostella* Frizzled receptors (see Fig. 1a) did not prevent endomesoderm specification and gastrulation in *Nematostella* despite completely aboralizing the ectoderm and endoderm of embryos ([19], see also Supplementary Figs. 6a[2] and 8). The latter result suggested that endomesoderm specification did not depend of Wnt/Fz/LRP5/6-mediated β-catenin signalling, while axial patterning did. Third, upon pharmacological activation of β-catenin signalling by GSK3β inhibitor treatment starting before 6 hours post-fertilization (hpf), *Nematostella* embryos failed to segregate the mesoderm, form blastopore lips and gastrulate[19]. Just like β-catenin morphants, these embryos remained spherical, however, these spheres expressed endodermal markers *Brachyury*, *FoxA* and *FoxB* in all cells, while mesodermal markers were abolished (Supplementary Fig. 6a[3]; [19,23]). In contrast, in the sea urchin, suppression of β-catenin signalling by *Axin* mRNA injection results in the ubiquitous expression of the anterior ectoderm markers (Supplementary Fig. 6b[1]; [26]), and overexpression of the GSK3β-insensitive variant of β-catenin strongly expands the endodermal portion of the embryo, however, importantly, without the loss of the mesoderm as evidenced by the presence of the mesodermally derived skeletogenic cells, pigment cells and *myosin heavy chain* expressing cells (Supplementary Fig. 6b[2]-f; [33]). Notably, overexpression of sea urchin *Wnt6* mRNA converted all of its ectoderm into endoderm without affecting the mesoderm – similar to the effect of GSK3β treatment after the specification of the endomesodermal domain in *Nematostella* (Supplementary Fig. 6a[4]; [19,34,67]). Taken

together, these data suggest that both in sea urchin and in *Nematostella*, endomesoderm and, subsequently, mesoderm specification is a cell-autonomous process, while endodermal fate can be induced in ectodermal cells by β-catenin signalling.

Our discovery of the vegetal nuclear β-catenin explains why mesodermal markers *SnailA, ERG* and others are ubiquitously expressed in the β-catenin morphants: they are de-repressed in the absence of the maternal β-catenin (see Supplementary Fig. 8). However, it does not explain why zygotic aboral/anterior ectoderm markers *Six3/6* and *FoxQ2a*, whose expression is also negatively regulated by Wnt/β-catenin signaling, are abolished[19,23], although we showed that anterior/aboral ectoderm identity represents the "default state" of the embryo[17] before endomesoderm gets specified. Recent analysis by Haillot et al.[31] suggests an answer to this question: *Six3/6* and *FoxQ2a* expression appears to be suppressed by MAPK signaling, which, in turn, is necessary for the mesodermal fate. Simultaneous suppression of β-catenin and MAPK signaling results in the ubiquitous expression of *Six3/6* and *FoxQ2a*, i.e. in the reversal to the "default" anterior ectoderm state.

The lack of β-catenin signalling at the future oral side of the early embryo is in line with our observation that mesoderm formation and gastrulation is not controlled by Wnt/Frizzled/LRP5/6-mediated signalling[19]. Moreover, early aboral nuclear β-catenin does not seem to depend on Frizzled /LRP5/6-mediated signalling either, since *LRP5/6* RNAi does not affect aboral nuclear β-catenin at 8 hpf (Supplementary Fig. 8), although we showed previously that *LRP5/6* RNAi completely depletes maternally deposited *LRP5/6* mRNA by 6 hpf[19], and no maternal LRP5/6 protein has been detected in the *Nematostella* zygotes by mass spectrometry[68]. Finally, the fact that β-catenin inhibits endomesoderm and, subsequently, mesoderm specification rather than activates it explains why upon treatment with a GSK3β inhibitor prior to 6 hpf, the whole embryo acquires endodermal rather than mesodermal or mixed endodermal and mesodermal fate[19,23]: ectoderm can be induced to become endoderm by β-catenin signaling, while endomesoderm and, subsequently, mesoderm is specified maternally in a maternal nuclear β-catenin-negative domain.

Importantly, our data, as well as the findings in the new study of Haillot et al.[31] suggest that the competence of specific genes in specific cells to be activated by β-catenin changes over time. Initially, maternal β-catenin activates the ectodermal (=non-endomesodermal) cell fate and represses the endomesodermal cell fate. In situ hybridization and qPCR analyses show that mesodermal genes react very strongly to the modulations of the β-catenin signaling intensity in 8-12 hpf embryos (see Supplementary Fig. 7 and [31]). In contrast, endodermal genes are either suppressed by or unresponsive to β-catenin at this stage (Supplementary Fig. 7), and become activated in the endomesodermal domain or in the definitive endoderm by another signal, most likely by Notch[31]. As the embryo develops, maternal nuclear β-catenin disappears, an O-A gradient of zygotic nuclear β-catenin forms, and the endodermally and ectodermally expressed genes, which we know to be activated by Wnt/β-catenin signaling from previous studies, fall under β-catenin control. In the future, it will be important to understand the nature of this switch in the regulation.

Supplementary references

66. Nocente-McGrath, C., McIsaac, R. & Ernst, S. G. Altered cell fate in LiCl-treated sea urchin embryos. *Dev. Biol.* **147,** 445-450 (1991).

67. McClay, D. R., Croce, J. C. & Warner, J. F. Conditional specification of endomesoderm. *Cells Dev.* **167,** 203716 (2021).

68. Levitan, S. et al. The making of an embryo in a basal metazoan: proteomic analysis in the sea anemone *Nematostella vectensis. Proteomics* **15,** 4096-4104 (2015).

Supplementary Tables

Supplementary table 1. qPCR primers

Bra_qF	CGCACTCAGCTTACTCCCAA
Bra_qR	AGGTCGATGACTTCGGATGC
FoxA_qF	GCCATGGGTATGGCAGGTAT
FoxA_qR	TGAAGTGCATGGGGTCGTAG
FoxB_qF	AAACAGTTACGGCAGCGCTAA
FoxB_qR	GGGAAAATGGTCCATGATGA
Six3/6_qF	TTCTTTGGTCCTTGCCTGTGGC
Six3/6_qR	TCGCTTGCAGTTTAGCGTGC
FoxQ2a_qF	CTGCCATTTCCACCATGTTACGCC
FoxQ2a_R	GTTGGCCTGCATCTTGCTCTCTTC
Sp1/4_qF	GCATGGAAAAACTCGTCCACT
Sp1/4_qR	TTCCAGTTCCTCGCCAATGC
DuxABC_qF	CGATTATCACAGTGTGCCTTC
DuxABC_qR	TGCCAGGAAATCGCTCGTACT
ERG_qF	TGTTCAGGATACACTGCTGG
ERG_qR	GGTTCTTCGAGTTCCGCTTC
Tbx19qF_GR	GCCTAAAGAACATCCCGCCA
Tbx19qR_GR	CCGGGAAGAGATGTTGCTGT
Wnt1_qF	CGAGCTTATCCAGTACATCGC
Wnt1_qR	GAGAATCTTGCCAAACACGTTC
Wnt3_qF	TGGACATGGTCTGGCTGTAA
Wnt3_qR	CTCAAGTCACCACCCCTCTC
WntA_qF	CAGACTAAAGACGAGGCGAGT
WntA_qR	GCCTCGTTGTTATGCTTCACC
qGAPDH_F_GR	TATGACTCCACCCATGGCA
qGAPDH_R_GR	GTGAAGACGCCAGTGGACTC

Supplementary table 2. HCR probes for *Nematostella ERG*

1_1_B2_ERG	CCTCGTAAATCCTCATCAaaATATATAATAAAAATATGAAGAACA
1_2_B2_ERG	CAAGTTCAAATATATTTATTTACAAaaATCATCCAGTAAACCGCC
2_1_B2_ERG	CCTCGTAAATCCTCATCAaaTTAGTTTTCATGCTGAACTTTAGCT
2_2_B2_ERG	ACATTATAAATTATCTATTTGGTACaaATCATCCAGTAAACCGCC
3_1_B2_ERG	CCTCGTAAATCCTCATCAaaCTATGAACAACAAAGTGTTTTCTTG
3_2_B2_ERG	AAAAACTCTTCGTAATACATAAATAaaATCATCCAGTAAACCGCC
4_1_B2_ERG	CCTCGTAAATCCTCATCAaaAAACAGCCGATGTCAATCATCATTT
4_2_B2_ERG	TTTCTTTAAAATTGTTTTGCTGTAAaaATCATCCAGTAAACCGCC
5_1_B2_ERG	CCTCGTAAATCCTCATCAaaTAGTCAATTGCCAACCATTACCATT
5_2_B2_ERG	TTTTTAATTTATATATAAAATACTAaaATCATCCAGTAAACCGCC
6_1_B2_ERG	CCTCGTAAATCCTCATCAaaGCTACCATTTTTACAAAAATATGCG
6_2_B2_ERG	TTTTTAAATCCTACATTGATTCCAGaaATCATCCAGTAAACCGCC
7_1_B2_ERG	CCTCGTAAATCCTCATCAaaAAACAGAGCTCCCACAGTGTGTAAA
7_2_B2_ERG	AAAAACAACTTCATTGATGGCTCTCaaATCATCCAGTAAACCGCC
8_1_B2_ERG	CCTCGTAAATCCTCATCAaaTATGATGTATATGTTTATAAACTAT
8_2_B2_ERG	ATAAATAGTCTCGATTCAATATATAaaATCATCCAGTAAACCGCC
9_1_B2_ERG	CCTCGTAAATCCTCATCAaaGCGTAGTAGGTCATACTGGCCATGG
9_2_B2_ERG	TAAAATGTCTTTGTGGGGTGGATTTaaATCATCCAGTAAACCGCC
10_1_B2_ERG	CCTCGTAAATCCTCATCAaaAAGCAGGTGGGGCACGCCCCCAGTA
10_2_B2_ERG	AGGGGTACATCGGTCGGGTCTGTGGaaATCATCCAGTAAACCGCC
11_1_B2_ERG	CCTCGTAAATCCTCATCAaaGTGCGAGTACAGCGATGCGGTCGGG
11_2_B2_ERG	CATCTGGAAGCAGGTGTTTGCGCCGaaATCATCCAGTAAACCGCC
12_1_B2_ERG	CCTCGTAAATCCTCATCAaaTTGTAAGCGGCCTGAGCATGCGCCT
12_2_B2_ERG	CCCGCGTAGAAGTCGGAGCCTGGGAaaATCATCCAGTAAACCGCC
13_1_B2_ERG	CCTCGTAAATCCTCATCAaaGCGCAAGGCCCTGGAAGTCGTACTT
13_2_B2_ERG	CGTCAGATGTGATTGGCTGGTTGAGaaATCATCCAGTAAACCGCC
14_1_B2_ERG	CCTCGTAAATCCTCATCAaaCATGATGTTTTTGTCGTAGTAGTAC
14_2_B2_ERG	GGCGTAGCGCTTGCCGTGGATTTTGaaATCATCCAGTAAACCGCC
15_1_B2_ERG	CCTCGTAAATCCTCATCAaaCCTCATCCGGGTCCACCAGTTTGAA
15_2_B2_ERG	TTTTTCTCTCTCCCCACCTTCTTGCaaATCATCCAGTAAACCGCC
16_1_B2_ERG	CCTCGTAAATCCTCATCAaaGTTAGCGTTCTTGGGGTCTGAGAGC
16_2_B2_ERG	GCCGTTGGTGCCCTCCCAGGCGATGaaATCATCCAGTAAACCGCC
17_1_B2_ERG	CCTCGTAAATCCTCATCAaaTGGCCGCTACCTGTAGCAGGGGTAT
17_2_B2_ERG	TCTAAGAGGAACTGCCATAGCTGAAaaATCATCCAGTAAACCGCC
18_1_B2_ERG	CCTCGTAAATCCTCATCAaaATGAGCCTGTCTGTACTGTTGCCTC
18_2_B2_ERG	CACGGTGGGGCATCGGGCTGGCATTaaATCATCCAGTAAACCGCC
19_1_B2_ERG	CCTCGTAAATCCTCATCAaaCTTCATGTGGGAGAGCCTGGGGGTT
19_2_B2_ERG	GACGGTCTTGTTCGGGACAACCCCGaaATCATCCAGTAAACCGCC
20_1_B2_ERG	CCTCGTAAATCCTCATCAaaGCGCGGTCGTAGGCATCGTTATTGC
20_2_B2_ERG	GATGCTGGGGCGCTCCAGGTCGGTGaaATCATCCAGTAAACCGCC
21_1_B2_ERG	CCTCGTAAATCCTCATCAaaTGGATGAGGCAGCGTTAGGGTTGCT
21_2_B2_ERG	AGCGCGACATGCTGTAGTCGGTACTaaATCATCCAGTAAACCGCC
22_1_B2_ERG	CCTCGTAAATCCTCATCAaaGAGGGATGAGGTAGGGGTGTAAGCA
22_2_B2_ERG	GATTACAGGGTGTGGTCTCGAGTCGaaATCATCCAGTAAACCGCC

23_1_B2_ERG	CCTCGTAAATCCTCATCAaaGCGAGAGCCGAGTCGATGTCGTCTG
23_2_B2_ERG	CAGGATTGGGTCGAGACTGAAGCGGaaATCATCCAGTAAACCGCC
24_1_B2_ERG	CCTCGTAAATCCTCATCAaaAGACGCACAGATGGGCCATCAGAAT
24_2_B2_ERG	TGAGATTCGGTAACGGTGTTTTTCTaaATCATCCAGTAAACCGCC
25_1_B2_ERG	CCTCGTAAATCCTCATCAaaGAAATCGTCTCGTGTGAGTCGGCAG
25_2_B2_ERG	GCCGTTGTAGGCTGGGGCGAGCTTCaaATCATCCAGTAAACCGCC
26_1_B2_ERG	CCTCGTAAATCCTCATCAaaATGTCGATGTCTTTAAGTGAGAATT
26_2_B2_ERG	TCGCGTCCATCGATGTTGAATCGGTaaATCATCCAGTAAACCGCC
27_1_B2_ERG	CCTCGTAAATCCTCATCAaaCGTGTTCTTTGGTCCAGCATAGAGG
27_2_B2_ERG	TGATTGCCCAAAGGATCCACTGGCGaaATCATCCAGTAAACCGCC
28_1_B2_ERG	CCTCGTAAATCCTCATCAaaATGAGCCAAGCTTTGTGGGGCAGGA
28_2_B2_ERG	GGCTGGGACGATGACTCTTCGGTTAaaATCATCCAGTAAACCGCC
29_1_B2_ERG	CCTCGTAAATCCTCATCAaaTTTGGCACGAATGCTCGTTTCGCCG
29_2_B2_ERG	GGAGAATTTCGCTCGAGATAATCGTaaATCATCCAGTAAACCGCC
30_1_B2_ERG	CCTCGTAAATCCTCATCAaaGTTTCTTGTAGTAGGAGTCGGTTTT
30_2_B2_ERG	GTTCTTCGAGTTCCGCTTCGAGTGGaaATCATCCAGTAAACCGCC
31_1_B2_ERG	CCTCGTAAATCCTCATCAaaGTGAAAAGACGAGTCGAAGAGTGAT
31_2_B2_ERG	GTTGGGTTTTACTGGTTTGTACGCGaaATCATCCAGTAAACCGCC
32_1_B2_ERG	CCTCGTAAATCCTCATCAaaACCGACGCATCGGGAACAACATTCG
32_2_B2_ERG	TCTTCCAGCAGTGTATCCTGAACATaaATCATCCAGTAAACCGCC
33_1_B2_ERG	CCTCGTAAATCCTCATCAaaATCTGTAACAATTCTCGGTGTGCGT
33_2_B2_ERG	CTGAACTTAAACCATACATCTTGTCaaATCATCCAGTAAACCGCC
34_1_B2_ERG	CCTCGTAAATCCTCATCAaaGGAATGCGACTGGTATAATTCCTTC
34_2_B2_ERG	AAGTAGTCAAAATTGTCTTTCTGTAaaATCATCCAGTAAACCGCC
35_1_B2_ERG	CCTCGTAAATCCTCATCAaaCGGAACAAATTTTCTTGTTCTGTCG
35_2_B2_ERG	CGATGTCAAATTTGTGATTATTCTTaaATCATCCAGTAAACCGCC
36_1_B2_ERG	CCTCGTAAATCCTCATCAaaTGTTTAGCGAATGATGCTCACGATC
36_2_B2_ERG	TTTTTGGGGGTGCTCTTCCTCGATGaaATCATCCAGTAAACCGCC
37_1_B2_ERG	CCTCGTAAATCCTCATCAaaAAATCAGTGCACCGACTCCGTGACA
37_2_B2_ERG	CTTACTCAAATTTATAGCACTAGCTaaATCATCCAGTAAACCGCC
