# Supplementary material for: β-catenin-driven endomesoderm specification is a Bilateria-specific novelty
Source: Nat Commun. 2025 Mar 12;16:2476. doi: 10.1038/s41467-025-57109-w (PMC11903683; doi:10.1038/s41467-025-57109-w)
Supplement: Supplementary file 4 — Supplementary Movie Legends [file 41467_2025_57109_MOESM4_ESM.docx]

**Supplementary movie 1. Live imaging of the sfGFP-β-catenin dynamics during development of the *Nematostella* embryo until the onset of gastrulation.** Example embryo 1. Note that nuclear sfGFP-β-catenin is visible in the interphase of every cell cycle until mid-blastula at the side opposite to where the mesoderm will form and start to invaginate.

**Supplementary movie 2. Live imaging of the sfGFP-β-catenin dynamics during development of the *Nematostella* embryo until the onset of gastrulation.** Example embryo 2. Note that nuclear sfGFP-β-catenin is visible in the interphase of every cell cycle until mid-blastula at the side opposite to where the mesoderm will form and start to invaginate.

**Supplementary movie 3. Live imaging of the sfGFP-β-catenin dynamics during development of the *Nematostella* embryo upon GSK3β inhibition with 5 µM alsterpaullone.** Example embryo 1. Note that nuclear sfGFP-βcatenin is localized in all nuclei throughout the embryo and keeps appearing in every cell cycle until the end of the movie, although the we filmed alsterpaullone-treated embryos for 1 hour longer than the untreated embryos shown in the Supplementary Movies 1 and 2. Also note that, as previously reported 19,23, in the embryos incubated in GSK3β inhibitor from fertilization on, mesoderm does not form.

**Supplementary movie 4. Live imaging of the sfGFP-β-catenin dynamics during development of the *Nematostella* embryo upon GSK3β inhibition with 5 µM alsterpaullone.** Example embryo 2. Note that nuclear sfGFP-βcatenin is localized in all nuclei throughout the embryo and keeps appearing in every cell cycle until the end of the movie, although the we filmed alsterpaullone-treated embryos for 1 hour longer than the untreated embryos shown in the Supplementary Movies 1 and 2. Also note that, as previously reported 19,23, in the embryos incubated in GSK3β inhibitor from fertilization on, mesoderm does not form.
