# Supplementary material for: β-catenin-driven endomesoderm specification is a Bilateria-specific novelty
Source: Nat Commun. 2025 Mar 12;16:2476. doi: 10.1038/s41467-025-57109-w (PMC11903683; doi:10.1038/s41467-025-57109-w)
Supplement: Supplementary file 9 — Source Data [file 41467_2025_57109_MOESM9_ESM.zip › Description of the Source Data.docx]

## **Description of the Source Data**

Supplementary Data File 1.

**Normalized measurements of the nuclear anti-GFP signal used to generate the β-catenin-sfGFP profile on Fig. 1f.** Each text file contains measurements from one stained embryo. See file number for n.

Supplementary Data File 2.

**Normalized measurements of the NBT/BCIP staining after whole mount in situ hybridization with the probe against *Axin*, which were used to generate the *Axin* profile on Fig. 1f.** Each text file contains measurements from one stained embryo. See file number for n.

Supplementary Data File 3.

**Raw measurements of the nuclear anti-GFP signal and NBT/BCIP staining after whole mount in situ hybridization with the probes against *Axin*, *Brachyury*, *Wnt2*, and *Six3/6*, which were used to generate the profiles on Supplementary Fig. 2.** Subfolders are named according to the staining used. Within the subfolders, each text file contains measurements from one stained embryo. In situ probes and treatment conditions (DMSO, 1 µM ALP, 2.5 µM ALP, 5 µM ALP) are mentioned in the file names. See file number for n.

**Supplementary Data File 4.**

**qPCR data for mesodermal, endodermal and zygotic ectodermal markers plotted in the Supplementary Fig. 7.** First sheet contains the Ct values for all the samples and the calibration curve equations for all the primer pairs. Second sheet expression intensity values (in arbitrary units) after normalization against GAPDH. Third sheet contains t and p values for the two-tailed t-tests.
